# *N*^6^-Methyladenosine Level in Silkworm Midgut/Ovary Cell Line Is Associated With *Bombyx mori* Nucleopolyhedrovirus Infection

**DOI:** 10.3389/fmicb.2019.02988

**Published:** 2020-01-10

**Authors:** Xing Zhang, Yunshan Zhang, Kun Dai, Zi Liang, Min Zhu, Jun Pan, Mingtian Zhang, Bingyu Yan, Hanxue Zhu, Ziyao Zhang, Yaping Dai, Manman Cao, Yuchao Gu, Renyu Xue, Guangli Cao, Xiaolong Hu, Chengliang Gong

**Affiliations:** ^1^School of Biology and Basic Medical Science, Soochow University, Suzhou, China; ^2^Institute of Agricultural Biotechnology and Ecological Research, Soochow University, Suzhou, China

**Keywords:** *Bombyx mori*, midgut, m6A, *Bombyx mori* nucleopolyhedrovirus, virus infection

## Abstract

*Bombyx mori* nucleopolyhedrovirus (BmNPV) is one of the most serious pathogens in sericulture and causes huge economic loss annually. The roles of N6-methyladenosine (m6A) modification in silkworms following BmNPV infection are currently unclear. Here, methylated RNA immunoprecipitation with next-generation sequencing were applied to investigate the m6A profiles in silkworm midgut following BmNPV infection. A total of 9144 and 7384 m6A peaks were identified from the BmNPV-infected (TEST) and uninfected silkworm midguts (CON), respectively, which were distributed predominantly near stop codons. Gene Ontology (GO) and Kyoto Encyclopedia of Genes and Genomes (KEGG) analysis of common m6A peaks in nuclear genes revealed that these m6A-related transcripts were associated with crucial signaling pathways. Comparative transcriptome analysis showed that 1221 differential expressed m6A peaks were identified between TEST and CON, indicating that m6A modification is regulated following BmNPV infection. GO and KEGG pathway analysis of the differentially expressed m6A peaks showed their association with signal transduction, translation, and degradation. To understand further the effect of the m6A machinery on virus infection, expression levels of m6A-related genes were altered in silencing and overexpression experiments. Expression of viral structural protein VP39 was increased in BmN cells by siRNA-mediated depletion of methyltransferase-like (METTL) enzyme genes (BmMETTL3, BmMETTL14) and cytoplasmic YTH-domain family 3 (BmYTHDF3), while the reverse results were found after overexpression of the m6A-related enzymes in BmN cells. Overall, m6A modification might be a novel epigenetic mechanism that regulation BmNPV infection and interference with this mechanism may provide a novel antiviral strategy for preventing BmNPV disease.

## Introduction

Chemical modifications are critical to post-transcriptional gene regulation in eukaryotes (Nachtergaele and He, 2018). More than 100 different RNA modifications have been reported, but the N6-methyladenosine (m6A) modification is regarded as the most common internal form of modification of mRNAs and long non-coding RNAs in eukaryotes, as well as in the RNAs of nuclear-replicating viruses (Roundtree et al., 2017; Imam et al., 2018; Coker et al., 2019). m6A regulates numerous RNA biology events by various mechanisms such as mRNA stability (Wang et al., 2014; Xu et al., 2014), translation efficiency (Meyer et al., 2015; Wang et al., 2015), nuclear export (Zheng et al., 2013), expression and alternative splicing patterns (Zhao et al., 2014; Liu et al., 2017), protein/RNA interactions (Liu et al., 2015), miRNA biogenesis and X-chromosome inactivation (Louloupi et al., 2018), as well as sex determination in *Drosophila* (Kan et al., 2017). However, little is known about m6A effects on virus infection, especially in the lepidopteran insect, the silkworm *Bombyx mori*.

Transcriptome-wide m6A profiling was first carried out in mammals and >12,000 m6A methylation sites have been identified from human transcripts. These m6A methylation sites are specifically around stop codons, 3′-untranslated regions (UTRs) and within long internal exons. Moreover, these m6A sites are highly conserved between humans and mice (Dominissini et al., 2012; Meyer et al., 2012). More than one-third of zebrafish maternal mRNAs have been identified with m6A modifications (Zhao et al., 2017). Most sites respond to various stimuli and modulated sites have also been found to be dynamically regulated (Dominissini et al., 2012). m6A modification exhibits tissue-specific regulation and methylation site selection has been found in different brain regions and types of neural cells in mice (Chang et al., 2017). m6A modifications play important roles in the regulation of nutrient metabolism in porcine liver (He et al., 2017). These results indicate that these chemical modifications are common and important in a variety of biological processes.

In addition to m6A sites in vertebrates, many m6A modification events have also been found in invertebrates, including plants and insects. Investigation of m6A transcriptome-wide profiles in *Arabidopsis thaliana* has shown that the m6A modification occurs in the mRNA of plants, and these m6A sites are found around start codons, different from the m6A sites near the stop codons and within 3′-UTRs identified in mammals (Luo et al., 2014). The m6A modification is found in the nuclei as well as the chloroplasts and mitochondria of *Arabidopsis*, and the m6A motif sequences in the chloroplasts and mitochondria are similar to the nuclear motifs (Wang et al., 2017). m6A modification profiling in rice shows that the m6A modification has a preference toward start and stop codons. m6A modification is negatively correlated with the level of gene expression (Li Y. et al., 2014). m6A modification is abundant in *Trypanosoma brucei* (Liu et al., 2019) and *B. mori* (Li et al., 2019). These findings show that m6A is a conserved modification among different species. Recently, m6A modifications have also been found in the genomes of RNA viruses and transcripts of DNA viruses, which play crucial roles in the regulation of virus infection (Tan and Gao, 2018).

As a lepidopteran model insect, *B. mori* has been widely used to investigate numerous cell processes. *B. mori* nucleopolyhedrovirus (BmNPV) is an enveloped, circular double-strand DNA virus that enters *B. mori* larvae mainly through oral feeding of viral-polyhedron-contaminated mulberry leaves, after which it establishes primary infection in the midgut epithelium. BmNPV infection causes huge economic losses in sericulture annually. Although numerous studies of differential expression of mRNAs, miRNAs, and proteins during BmNPV infection have been carried out, the m6A modification profiles of mRNAs in the silkworm following BmNPV infection remain uninvestigated.

In the present study, methylated RNA immunoprecipitation with next-generation sequencing (MeRIP-Seq) was applied to determine the differential m6A transcriptome-wide map following BmNPV infection of the midgut in silkworm larvae. We show that the presence of thousands of m6A peaks in the transcriptome of the uninfected silkworm midgut, which were distributed predominantly near stop codons. m6A peaks in nuclear genes were associated with translation, signal transduction, degradation, transcription, and transport. Gene Ontology (GO) and Kyoto Encyclopedia of Genes and Genomes (KEGG) analysis of 1221 differentially expressed m6A peaks showed that m6A modification was altered following BmNPV infection, and that the altered m6A peaks were associated with important signaling pathways, such as signal transduction, translation, and degradation. In addition, expression of VP39 was increased by silencing of BmMETTL3, BmMETTL14, and BmYTHDF3 genes with siRNAs, while overexpression of the three genes of the m6A machinery decreased VP39 expression in BmN cells. Overall, m6A levels were affected by BmNPV virus infection, which indicated that m6A modification might be a novel epigenetic mechanism that regulates BmNPV infection and that interference with m6A modification could provide a novel antiviral strategy for preventing BmNPV disease.

## Materials and Methods

### Silkworm Maintenance, BmNPV Preparation, and Virus Infection

Larvae of the domesticated silkworm strain Jingsong × Haoyue were reared in our laboratory with fresh mulberry leaves at 25°C. BmNPV was propagated in silkworms and the polyhedrons were collected by centrifugation at 8000 rpm. One million polyhedrons from BmNPV were evenly coated on the mulberry leaves with in an area of 4 × 4 cm (length ^∗^ width). Fifth instar silkworm larvae (on 3rd day after molt) were fed on these leaves. Larvae at the same instar stage fed on untreated leaves were used as the control group.

### Midgut Dissection, Total RNA Extraction, and Sequencing

Thirty normal healthy silkworms and 30 BmNPV-infected silkworms were collected at 72 h after feeding for midgut dissection. Total RNAs were extracted from normal midguts and BmNPV-infected midguts with TRIzol reagent (Invitrogen, Carlsbad, CA, United States) and mRNAs with polyA were enriched with Oligo-dT. The complete mRNA sequences were disrupted with fragmentation buffer (10 mM ZnCl_2_, 10 mM Tris–HCl pH 7.0). The obtained fragments were divided into two parts: one was captured with m6A antibody (ab151230; Abcam, Cambridge, MA, United States) for enrichment of mRNA with m6A modification and the other was used as input for transcriptomic library construction. The fragments collected from the m6A antibody (ab151230; Abcam, Cambridge, MA, United States) were also used in parallel for transcriptomic library construction. The two constructed libraries were used for sequencing with Illumina Hiseq 4000 at Shanghai OE Biotech. Co. Ltd. The raw data were submitted to the Sequence Read Archive with accession numbers (SRR10141250, SRR10141249, SRR10141248, and SRR10141247).

### Data Analysis

Raw data (raw reads) in fastq format were first processed using Trimmomatic software (Bolger et al., 2014). Clean data (clean reads) were obtained by removing reads containing adapters, reads containing poly-*N*, and low-quality reads from the raw data. Then, 250,000 paired reads were randomly extracted from the clean data for alignment with the NT database^1^ using blastn software, and those reads with *e* < 1 × 10^–10^ and >80% coverage were collected for further analysis. rRNA reads were removed using SortMeRNA software (Kopylova et al., 2012). After discarding the rRNA reads, the remaining clean reads were mapped to the reference genome (*B. mori* assembly ASM15162v1) using HISAT2 with default parameters (Kim et al., 2015). Unique reads with high mapping quality were retained, and potential polymerase chain reaction duplicates were marked with Picard^2^.

m6A-seq data quality was evaluated with the Trumpet R package (Zhang et al., 2018). The m6A-enriched peaks in each m6A immunoprecipitation sample were identified by MeTDiffpeak calling software (Cui et al., 2018) (parameters: “FRAGMENT_LENGTH=200, PEAK_CUTOFF_PVALUE = 0.05”), with the corresponding input sample serving as a control. Called peaks were annotated by intersection with gene architecture using ChIPseeker software (Yu et al., 2015). The differential expression of transcripts with m6A methylome between BmNPV-infected and uninfected midguts was detected with MeTDiff with the following parameters: “FRAGMENT_LENGTH=200, PEAK_CUTOFF_PVALUE = 0.05.” The differential peaks were annotated by ChIPseeker software. GO enrichment and KEGG pathway enrichment analysis of peaks and differential peaks were performed using R based on the hypergeometric distribution.

### Analysis of m6A Machinery Genes

The cellular m6A machinery includes proteins that act as writers, erasers, and readers of m6A (Gokhale et al., 2016). In mammals, RNA m6A methylation is catalyzed by a polyprotein complex composed of methyltransferase-like (METTL) enzymes METTL3, METTL14, and other factors. The cytoplasmic YTH-domain family (YTHDF)1, YTHDF2, and YTHDF3 proteins bind to m6A through their C-terminal YTH domain (Gokhale et al., 2016). METTL3 (NM_019852.4), METTL14 (NM_020961.3), and YTHDF2 (NM_001172828.1) of humans were blasted to the silkworm EST database to predict the writers and readers of the m6A machinery. The domain analysis of writers and readers from the silkworm was conducted with the SMART database^3^. The molecular weight was calculated with Compute pI/Mw^4^.

### Transient Expression of m6A Machinery Genes

The pIZT-V5-his plasmid (Invitrogen) was used to overexpress BmMETTL3 (XM_012695409.2), BmMETTL14 (XM_004924348.3), and BmYTHDF3 (XM_004924548.3) genes in BmN cells. Sequences of the ORFs for the recombinant plasmids pIZT-V5-his-BmMETTL3 (*Eco*RI/*Sac*II), pIZT-V5-his-BmMETTL14 (*Kpn*I/*Sac*II), and pIZT-V5-his-BmYTHDF3 (*Eco*RI/*Xba*I) were inserted into the indicated restriction sites of the pIZT-V5-his vector, respectively. BmN cells (1.5 × 10^5^ cells/well) were seeded in six-well plates and cultured overnight at 28°C. Transfection was performed using Lipofectamine^®^ 3000 Transfection Reagent (Invitrogen). Briefly, 2 μg recombinant plasmid was mixed with the transfection reagent gently and then added to a 3-cm dish with 2 ml culture medium without 10% fetal calf serum. Four hours later, the medium was replaced with complete medium and cultured at 28°C. Expression levels of BmMETTL3, BmMETTL14, and BmYTHDF3 genes in transfected cells were detected with real-time PCR assay.

### siRNA-Mediated Silencing of the m6A Machinery Genes

According to the mRNA sequences of BmMETTL3, BmMETTL14, and BmYTHDF3, specific siRNA-1/2/3 of BmMETTL3, BmMETTL14, and BmYTHDF3 genes and non-specific siRNA-NC as a negative control were designed and synthesized by Shanghai Integrated Biotech Solutions (Table 1). BmN cells (1.5 × 10^5^ cells/well) were seeded in six-well plates and cultured overnight at 28°C. Transfection was performed using Lipofectamine^®^ 3000 Transfection Reagent (Invitrogen). Briefly, 2 μg siRNA was mixed with the transfection reagent gently and then added to a 3-cm dish with 2 ml culture medium without 10% fetal calf serum. Four hours later, the medium was replaced with complete medium and cultured at 28°C. The non-specific siRNA-NC (20 nM) was used as the control. BmN cells were subsequently treated using the same procedure as mentioned above for the transient expression experiments. Each transfection was repeated three times. Expression levels of BmMETTL3, BmMETTL14, and BmYTHDF3 genes were detected with real-time PCR to evaluate the extent of gene depletion following siRNA treatment.

**TABLE 1:** siRNA sequences.

**Target gene**		**Sense**	**Anti-sense**
METTL3 (LOC101745362)	LOC101745362-2123	GGUCAUCGUUCUAAACUAACC	UUAGUUUAGAACGAUGACCGG
METTL14 (LOC101738928)	LOC101738928-1366	GCUUCUUCUACAAUACAUAUU	UAUGUAUUGUAGAAGAAGCUG
YTHDF3 (LOC101736882)	LOC101736882-1528	GAUUAUAAUUCCAAUUCUAGC	UAGAAUUGGAAUUAUAAUCUA
NC	NC-274	GGCUACGUCCAGGAGCGCACC	UGCGCUCCUGGACGUAGCCUU

### Cell Viability

BmN cells were cultured for 12 h in 96-well plates before treatment with different siRNAs targeting BmMETTL3, BmMETTL14, BmMETTL3+14, and BmYTHDF3. For a period of 60 h, the treated cells were collected for cell viability detection. Another group of BmN cells was transfected with the recombinant plasmids pIZT-V5-his-BmMETTL3, pIZT-V5-his-BmMETTL14, or pIZT-V5-his-BmYTHDF3. At Forty-eight hours post-transfection, the treated cells were collected for cell viability detection. Cell viability was estimated with the3-(4,5-dimethyl-2-thiazolyl)-2,5-diphenyl-2-H-tetrazolium bromide (MTT) assay. Absorbance was detected at 490 nm with a reference wavelength of 595 nm.

### BmNPV Infection Assay

Budded virus containing EGFP-tagged BmNPV (BmNPV-GFP) was kindly gifted by Professor Xiaofeng Wu, Zhejiang University. At 48 h post-transfection, BmNPV-GFP [multiplicity of infection (MOI) = 5] was used to infect siRNA- and plasmid-transfected cells. At 48 h post-infection, the cells were harvested for western blotting with BmNPV structural protein VP39 antibody (gifted by Professor Zhongjian Guo, Jiangsu University) as the primary antibody. α-Tubulin was used as the reference protein, which was detected with anti-TUBB1 mouse monoclonal antibody (Abcam). Goat anti-mouse IgG H&L (Abcam) was used as the secondary antibody. Quantitative analysis of the visible bands from western blotting was performed using the ImageJ program. The integrated density values of the bands corresponding to the detected proteins were normalized to α-tubulin.

### Real-Time PCR

The expression levels of BmMETTL3, BmMETTL14, and BmYTHDF3 mRNAs were detected in the cells transfected with expression plasmids or siRNAs with real-time PCR. The procedure for real-time PCR assay was carried out according to the protocol supplied by the manufacturer (Bio-Rad, United States). The expression level of mRNAs was normalized to TIF-4A gene as the internal standard control and calculated using the 2^–ΔΔCt^ method (Livak and Schmittgen, 2001). All experiments were repeated in triplicate. The primers are listed in Supplementary Table 1.

## Results

### Validation of BmNPV Infection in *B. mori*

**FIGURE 1:**
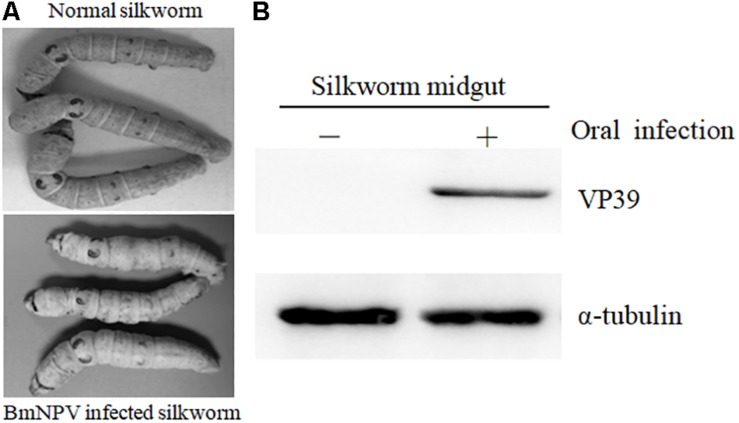
Validation of BmNPV infection in *B. mori*. **(A)** Typical pathological symptoms of BmNPV-infected *B. mori.* **(B)** Detection of viral structural protein VP39 with western blotting.

MeRIP-Seq was used to analyze the m6A modification in the transcriptome of the midguts in 6-day-old fifth instars silkworm larvaes. BmNPV infection was confirmed via observation of typical pathological symptoms, such as swelling of the larvae. In addition, viral structural protein VP39 was detected in the BmNPV-infected midguts but not in uninfected midguts (Figure 1). These findings indicate that *B. mori* was infected with BmNPV and could be used for further study.

### Transcriptome-Wide Detection of m6A Modification in BmNPV-Infected and Uninfected Midguts

Sequencing data were further analyzed by Hisat2 software (Kim et al., 2015), which was used to align the clean reads to the reference genome of *B. mori* (assembly ASM15162v1) and acquire genetic information for the m6A modification. A total of 49,934,564 and 62,659,272 reads were identified from uninfected and BmNPV-infected midguts, respectively. After filtering out low-quality data, 36,054,610 (72.20% of the total reads) and 49,054,967 (78.29% of the total reads) high-quality reads were mapped to the reference genome of *B. mori* (assembly ASM15162v1). Among the mapped reads, 32,838,534 (65.76%) and 43,853,738 (69.99%) were uniquely mapped to the genome and 13,188,800 (26.41%) and 17,221,827 (27.48%) were mapped to splice reads, respectively (Table 2).

**TABLE 2:** The statistics of sequencing data.

**Sample**	**midgut_input**	**midgut_BmNPV_input**	**midgut_BmNPV_IP**	**midgut_IP**
Total reads	54987860	57547900	62659272	49934564
Total mapped reads	38103322 (69.29%)	43762914 (76.05%)	49054967 (78.29%)	36054610 (72.20%)
Multiple mapped	3706223 (6.74%)	4873745 (8.47%)	5201229 (8.30%)	3216076 (6.44%)
Uniquely mapped	34397099 (62.55%)	38889169 (67.58%)	43853738 (69.99%)	32838534 (65.76%)
Read-1	17278280 (31.42%)	19530063 (33.94%)	22057079 (35.20%)	16510803 (33.06%)
Read-2	17118819 (31.13%)	19359106 (33.64%)	21796659 (34.79%)	16327731 (32.70%)
Reads map to “+”	17192864 (31.27%)	19449225 (33.80%)	21931093 (35.00%)	16410808 (32.86%)
Reads map to “−”	17204235 (31.29%)	19439944 (33.78%)	21922645 (34.99%)	16427726 (32.90%)
Non-splice reads	21503641 (39.11%)	24050026 (41.79%)	26631911 (42.50%)	19649734 (39.35%)
Splice reads	12893458 (23.45%)	14839143 (25.79%)	17221827 (27.48%)	13188800 (26.41%)
Reads mapped in proper pairs	33686974 (61.26%)	38067266 (66.15%)	42392836 (67.66%)	31929954 (63.94%)

**FIGURE 2:**
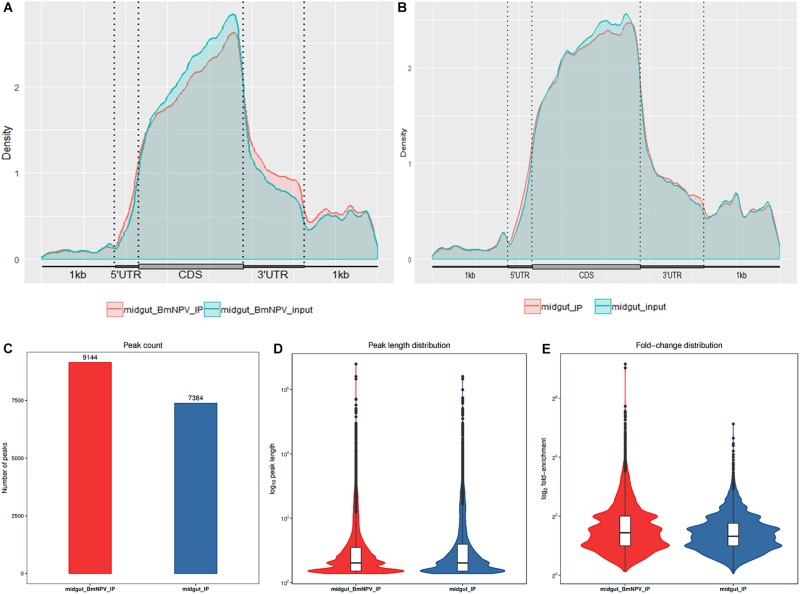
Characterization of m6A peaks in the transcriptome of BmNPV-infected and uninfected midgut. **(A)** m6A sites that appeared on the transcripts in BmNPV-infected midgut. **(B)** m6A sites that appeared on the transcripts in uninfected midgut. **(C)** Number of m6A peaks identified in BmNPV-infected and uninfected midgut. **(D)** Comparison of the distribution of m6A peaks between BmNPV-infected and uninfected midgut. **(E)** Fold change distribution of m6A peaks between BmNPV-infected and uninfected midgut.

To validate the efficiency of the RIP-seq data, R-package-Trumpet was used for quality control and to confirm the reliability of the experimental results (Zhang et al., 2018). The quality of m6A-seq data was shown to be sufficient. The enrichment analysis of m6A peaks on mRNAs showed that most peaks were distributed on the 3′-end of mRNAs (Figures 2A,B). Analysis of the sequencing data of immunoprecipitates with m6A antibody identified 7384 and 9144 peaks in samples from uninfected midguts and BmNPV-infected midguts, respectively (Figure 2C). The average length of the peaks was 869.77 and 656.94 bp in uninfected and BmNPV-infected midguts, respectively. The results suggest that the transcriptome of BmNPV-infected midgut, which had more A sites compared to the uninfected samples, had been modified by the related methyltransferase enzymes of the m6A machinery.

The length and the enrichment multiple distribution of m6A methylation peaks were analyzed using the RNA sequences with m6A sites. The length of mRNA with m6A sites from BmNPV-infected midgut was similar to that of uninfected midgut (Figure 2D), while the enrichment multiple distribution analysis showed that more reads were enriched in the peaks of the BmNPV-infected midgut than in those of the uninfected midgut (Figure 2E). These results also demonstrated that more transcripts in the BmNPV-infected midgut may have been modified with the related methyltransferase enzymes from the m6A machinery.

### Distribution of the m6A Modification in the Midgut Transcriptome

**FIGURE 3:**
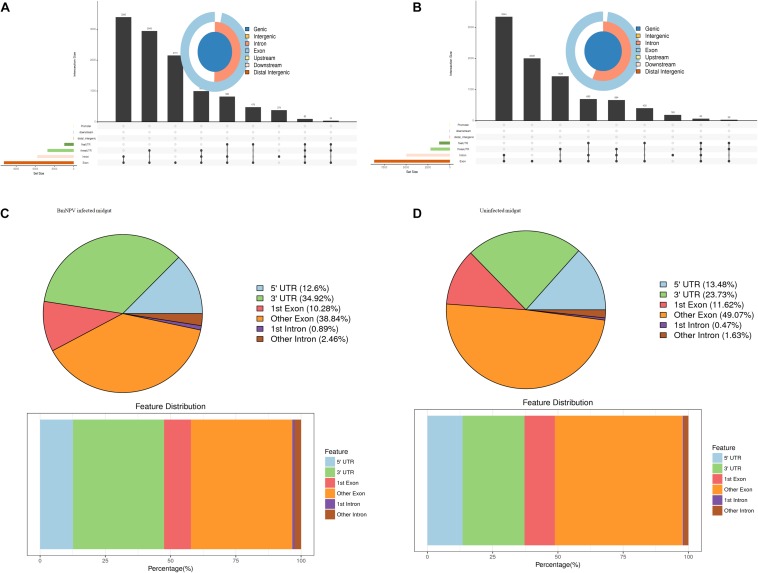
Distribution of m6A peaks on gene functional elements. **(A)** Distribution of m6A peaks on gene functional elements in BmNPV-infected midguts. **(B)** Distribution of m6A peaks on gene functional elements in uninfected midguts. **(C)** Transcriptome-wide distribution of m6A peaks in BmNPV-infected midguts. **(D)** Transcriptome-wide distribution of m6A peaks in uninfected midguts.

To understand the preferential location of the m6A modification in the transcripts, we investigated the localization of the m6A peaks in the gene functional elements of the entire transcriptome from both uninfected and BmNPV-infected midguts. The distribution of m6A peaks on gene functional elements showed that most of the peaks were distributed at the 3′-UTR and other exons in both BmNPV-infected and uninfected midguts (Figures 3A,B). Considering that the annotation results of gene elements corresponding to the peaks may overlap, we further analyzed the distribution of detected peaks on gene functional elements. The annotation results showed that most of the peaks appeared in the exons, introns, 5′-UTRs and 3′-UTRs (Figures 3C,D). These results indicated that the m6A modification is closely related to RNA expression and may have hotspot regions in the host transcripts.

### m6A-Containing Genes Are Associated With Important Biological Pathways

**FIGURE 4:**
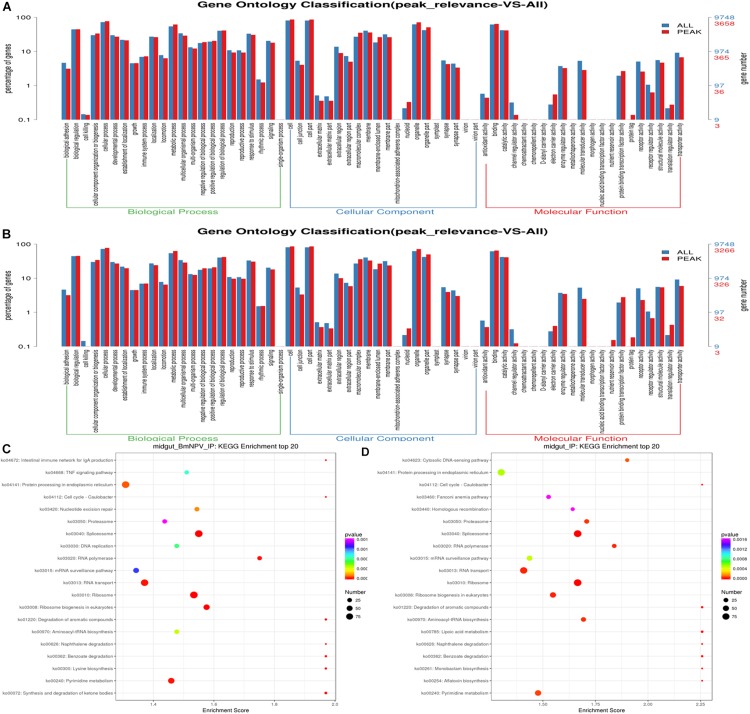
GO and KEGG analysis of m6A-containing transcripts. **(A)** GO analysis of m6A-containing transcripts in BmNPV-infected midguts. **(B)** GO analysis of m6A-containing transcripts in uninfected midguts. **(C)** Top 20 KEGG enrichments of m6A-containing transcripts in BmNPV-infected midguts. **(D)** KEGG classification of m6A-containing transcripts in uninfected midguts.

To explore the potential signaling pathways related to m6A-containing transcripts, GO analysis was carried out by Tritrydb online. A total of 305 IDs were uploaded to Tritrydb and analyzed with the GO Enrichment tool. Default settings were used; however, the results were limited to GO slim terms. The pathways related to m6A-genes were enriched in GO terms referring to binding (209), catalytic activity (109), transporter activity (14), protein binding transcription factor activity (11), molecular transducer activity (10), and structural molecule activity (9) in the molecular function category. Many genes were enriched in cellular process (239), metabolic process (176), biological regulation (157), regulation of biological process (149), cellular component organization or biogenesis (126), and response to stimulus (104) in biological process (Figures 4A,B). The pathways related to the mRNAs modified by m6A were enriched in spliceosome, ribosome, and RNA transport in BmNPV-infected midguts, while proteasome, RNA transport, and spliceosome were enriched in uninfected midguts (Figures 4C,D). These results indicate that the m6A modification may be closely related to gene expression and regulation.

### Differential m6A Methylation of mRNAs in Uninfected and BmNPV-Infected Midgut

**FIGURE 5:**
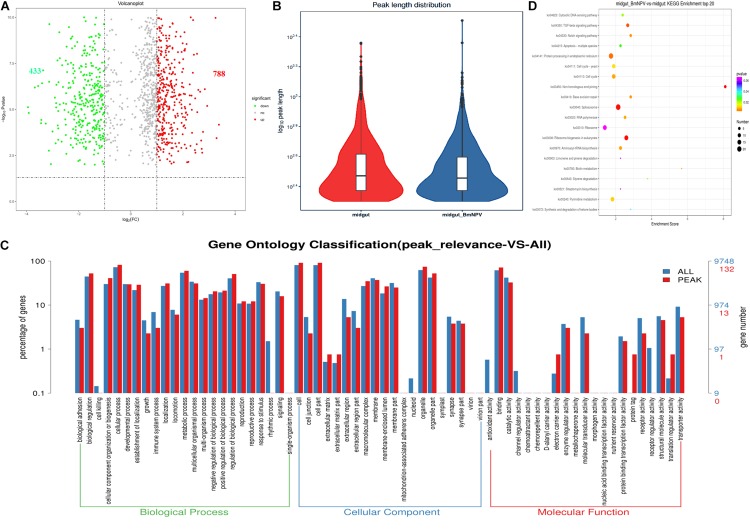
Analysis of sites with differential m6A modification following BmNPV infection. **(A)** Volcano plot of sites with differential m6A modification. **(B)** Distribution of sites with differential m6A modification. **(C)** GO analysis of transcripts with differential m6A modification. **(D)** Top 20 KEGG enrichment of transcripts with differential m6A modification.

To uncover the differential m6A methylation between uninfected and BmNPV-infected midgut, statistics were applied with threshold *p* < 0.05. We identified 1221 significantly differentially expressed m6A transcripts, of which 788 and 433 were upregulated and downregulated, respectively, during BmNPV infection. The distribution of the differentially expressed peaks on transcripts was nearly identical between the two groups (Figures 5A,B). Based on GO analysis of these differentially expressed m6A transcripts, the major molecular functions in these transcripts were related to binding activity, catalytic activity, structural molecular activity, molecular transducer activity, transporter activity, and enzyme regulator activity (Figure 5C). Based on KEGG pathway analysis transcripts with differential m6A modification associated with signal transduction and translation (Figure 5D). These results indicate that m6A methylation may play a crucial role in numerous RNA biology events and that the appearance of peaks of m6A in transcripts may be related to BmNPV infection.

### Prediction of m6A Machinery Components

Proteins that act as writers and readers of m6A were identified in the silkworm genome and subjected to bioinformatics analysis. Human enzymes METTL3 (NM_019852.4) and METTL14 (NM_020961.3) were blasted to the silkworm EST database, which resulted in the identification of *B. mori* N6-adenosine-methyltransferase 70 kDa subunit (XM_012695409.2) and *B. mori* METTL protein 14 homolog (XM_004924348.3) as candidate writers of the silkworm m6A machinery, named as BmMETTL3 and BmMETTL14, respectively. The human cytoplasmic YTHDF2 (NM_001172828.1) was also blasted to the silkworm EST database, and *B. mori* YTH domain-containing family protein 3 (XM_004924548.3) was predicted to be a reader of m6A machinery, named as BmYTHDF3.

**FIGURE 6:**
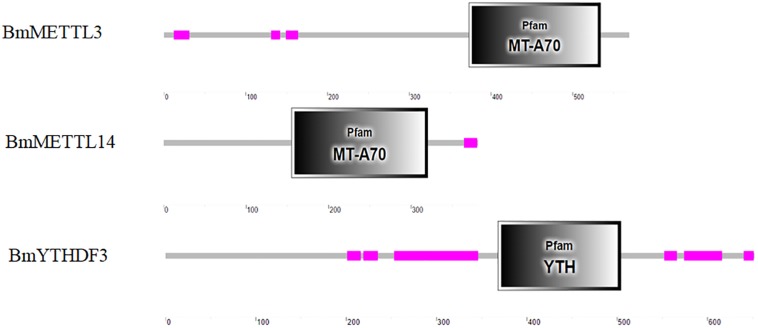
Analysis of conserved domains analysis of BmMETTL3, BmMETTL14, and BmYTHDF3 with the SMART software.

The *in silico* analysis showed that the coding sequences of BmMETTL3, BmMETTL14, and BmYTHDF3 were 1710, 1146, and 1959 bp corresponding to molecular weights of 62.98, 40.09, and 73.95 kDa, respectively. The specific domains in the m6A machinery components were predicted with the SMART database, which showed that BmMETTL3 and BmMETTL14 contained an MT-A70 domain, originally identified as the *S*-adenosylmethionine-binding subunit of human N6-adenosine-methyltransferase (MTase), an enzyme that sequence-specifically methylates adenines in pre-mRNAs. The YTH domain in BmYTHDF3 corresponds to an evolutionarily conserved m6A-dependent RNA binding domain (Figure 6). These domains from the three proteins in the m6A machinery were similar to those identified in other species, which confirmed that the m6A machinery is conserved across different species.

### Effect of the Depletion of the Methyltransferases and the m^6^A-Binding YTHDF Protein on Virus Infection

**FIGURE 7:**
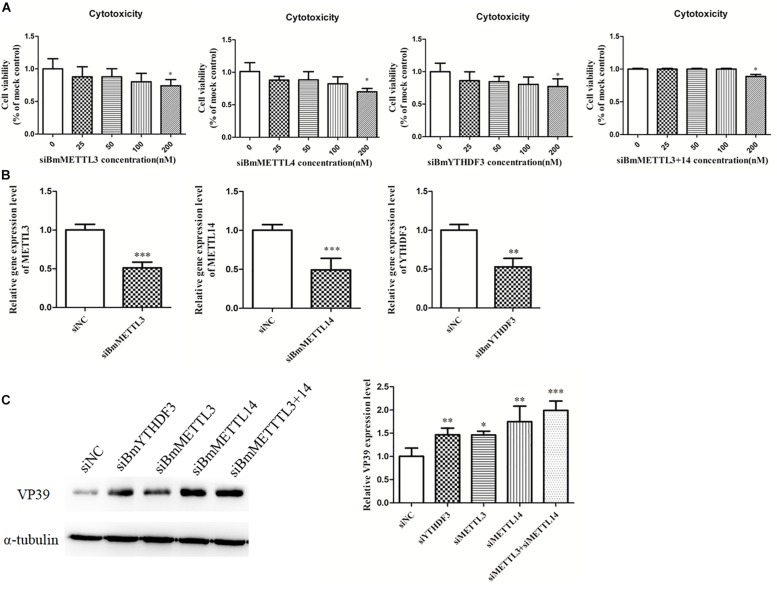
Depletion of BmMETTL3, BmMETTL14, and BmYTHDF3 expression levels with siRNAs and its effect on replication of BmNPV. **(A)** Viability assay of siRNA-treated cells. **(B)** The expression levels of BmMETTL3, BmMETTL14, and BmYTHDF3 gene in the siRNA-treated cells were detected with real-time PCR. **(C)** Effect of depletion of BmMETTL3, BmMETTL14, and BmYTHDF3 genes on VP39 expression. Statistically significant differences between the mean values were determined by Student’s *t*-test (^∗^*p* < 0.05, ^∗∗^*p* < 0.01, and ^∗∗∗^*p* < 0.001).

To detect the effect of the m6A modification of mRNAs on BmNPV infection, BmMETTL3 and BmMETTL14 and m6A reader protein BmYTHDF3 were depleted by siRNA in BmN cells and these cells were subsequently infected with BmNPV (MOI = 5). To understand whether the depletion of the m6A machinery genes had an adverse effect on cells, BmN cell viability was examined with the MTT assay and the results showed that depletion of BmMETTL3, BmMETTL14, BmMETTL3+14 (methyltransferase complex), and BmYTHDF3 by >50% did not impair the cells (Figures 7A,B). At 48 h post-infection, the infected cells were harvested and subjected to western blotting using VP39 antibody. Depletion of BmMETTL3, BmMETTL14, BmMETTL3+14, and BmYTHDF3 significantly increased expression of viral protein VP39, compared with cells treated with non-targeted control siRNA (Figure 7C).

### Effect of the Overexpression of the m^6^A Machinery Methyltransferases and the m^6^A-Binding YTHDF Protein on Virus Infection

**FIGURE 8:**
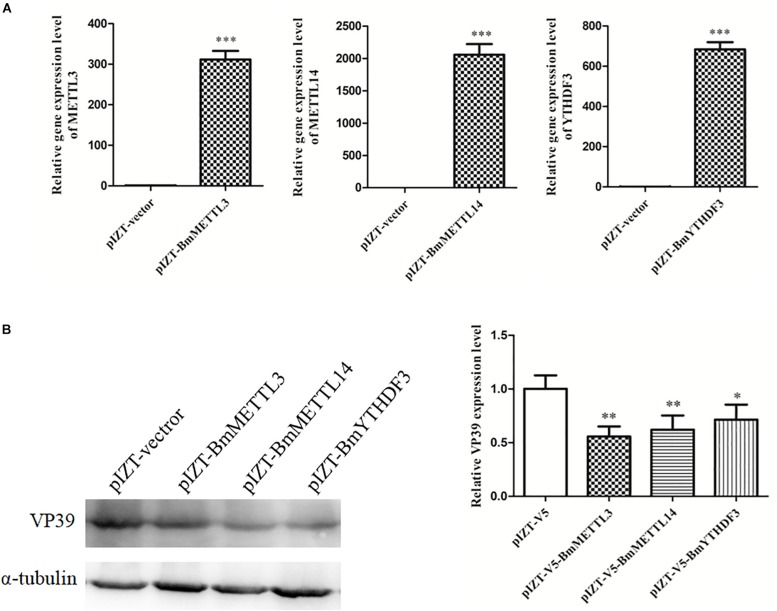
Overexpression of BmMETTL3, BmMETTL14, and BmYTHDF3 expression levels with recombinant plasmids and its effect on replication of BmNPV. **(A)** The expression levels of BmMETTL3, BmMETTL14, and BmYTHDF3 gene in the transfected cells were detected with real-time PCR. **(B)** Effect of overexpression of BmMETTL3, BmMETTL14, and BmYTHDF3 genes on VP39 expression. Statistically significant differences between the mean values were determined by Student’s *t*-test (^∗^*p* < 0.05, ^∗∗^*p* < 0.01, and ^∗∗∗^*p* < 0.001).

To detect the effect of m6A on BmNPV infection, BmMETTL3 and BmMETTL14 and m6A reader protein BmYTHDF3 were overexpressed by recombinant plasmids in BmN cells and these cells were infected with BmNPV (MOI = 5). The results showed that the expression levels of BmMETTL3, BmMETTL14, and BmYTHDF3 were significantly increased by recombinant pIZT-V5 vectors following transfection (Figure 8A), and that overexpression of these genes in BmN cells significantly decreased expression of VP39 (Figure 8B). These results demonstrated that the m6A machinery may play an important role in the process of BmNPV infection.

## Discussion

m6A methylation is a post-transcriptional modification that regulates numerous cellular processes by various mechanisms. We investigated the m6A profiles in the transcriptome of the *B. mori* midgut following BmNPV infection. The m6A modification was conserved in the *B. mori* transcriptome, similarly to other species. The m6A modification in the transcripts was affected by BmNPV infection, indicating that dynamic RNA modification events occur in cells in response to stimuli such as virus infection. Our findings provide novel clues to illuminate the mechanism of BmNPV infection at the mRNA modification level.

It is reported that many cellular processes are regulated by m6A modification, which could mediate mRNA degradation in numerous species. Therefore, abnormal m6A methylation levels in cells under stress might lead to dysfunction of RNA metabolism and cause corresponding diseases in mammals and other species (Zheng et al., 2013). METTL3, METTL14, and Wilms’ tumor 1-associated protein constitute the methyltransferase complex that carries out the m6A modification of nuclear RNAs (Liu et al., 2014; Ping et al., 2014). Fat mass and obesity-associated protein (FTO) and AlkB family member 5 act as RNA demethylases, which are responsible for removing the m6A modification in RNAs (Jia et al., 2011; Fu et al., 2014). Three host proteins (YTHDF1, 2, and 3) have been identified as selective m6A-binding proteins (readers) in mammalian cells (Dominissini et al., 2012; Wang and He, 2014; Wang et al., 2015). Three m6A machinery components were predicted in the silkworm and multialigned with similar components in other species. The m6A machinery in the silkworm is conserved with the machinery in other species, including vertebrates, plants, and yeast. Dysregulation of the m6A machinery was associated with development and physiological defects. The m6A level can be decreased by disruption of the *METTL3* homolog (*MTA*), which leads to arrest at the globular stage of embryo development in *Arabidopsis* (Zhong et al., 2008). In humans, *FTO* gene mutation results in a recessive lethal syndrome (Boissel et al., 2009). In mice, the loss of the *FTO* gene causes postnatal growth retardation, and *FTO* overexpression leads to increased food intake and obesity (Boissel et al., 2009). The m6A mRNA methylation also affects the mammalian circadian clock (Church et al., 2010). In this study, it was demonstrated that the m6A levels in the transcriptome of the midgut were changed following BmNPV infection. The expression of *BmMETTL3*, *BmMETTL14*, and *BmYTHDF3* genes was altered during BmNPV infection (data not shown), which may have caused the fluctuation in m6A levels in transcriptome of the cells. It is known that the m6A modification in mRNAs is found in most species and is enriched near the 3′-UTR and stop codon in mice and other mammals. Most m6A sites in mRNAs can respond dynamically to various stimuli (Dominissini et al., 2012). In our analysis of the m6A peak distribution on gene functional elements, we found that most of the peaks appeared at 3′-UTRs and that dynamically modulated m6A sites were found in the midgut transcriptome following BmNPV infection. These results indicate that the m6A peak distribution was similar to that in other species and that m6A sites in mRNAs responded to virus stimulation.

To understand further the roles of the m6A-containing transcripts in the midgut after BmNPV infection, the related transcripts were extracted and underwent GO annotation and KEGG pathways analysis. Based on GO annotation, we found that most of the m6A-containing mRNAs were enriched in the categories of molecular function, cellular process, and biological process. Differential m6A methylation was found in BmNPV-infected midguts, and differentially expressed transcripts containing m6A were analyzed. Based on GO analysis, the major molecular functions in these transcripts were relevant to binding activity, catalytic activity, structural molecular activity, molecular transducer activity, transporter activity, and enzyme regulator activity. Based on KEGG pathway analysis, transcripts with differential m6A modification were related to signal transduction and translation. These results indicated that the m6A modification not only occurs in insects, but also that its pattern changes during virus infection.

Changes in m6A modification occurred following BmNPV infection, suggesting that these altered transcripts with m6A may be related to the process of BmNPV infection. We investigated the role of BmMETTL3, BmMETTL14, and BmYTHDF2 genes during replication of BmNPV. The expression of these genes was silenced with corresponding siRNAs, which showed that replication of BmNPV was increased following depletion of BmMETTL3, BmMETTL14, and BmYTHDF3 genes in BmN cells. Conversely, replication of BmNPV was inhibited by overexpression of BmMETTL3, BmMETTL14, and BmYTHDF3 genes in BmN cells. These results indicate that the components of the m6A machinery respond to BmNPV infection and that the process of BmNPV infection can be altered by overexpression and siRNA-mediated silencing of the genes related to the m6A machinery. We speculate that the effect of m6A on BmNPV infection can be attributed to the altered function of m6A methyltransferases on viral or host mRNA. The m6A-binding YTHDF proteins that specifically recognize the m6A modification via a conserved m6A-binding pocket in the YTH domain can modulate m6A-modifed mRNA stability and translation (Sheth and Parker, 2003; Fu et al., 2014; Li F. et al., 2014; Shi et al., 2017). These results suggest that endogenous Y1-3 reader proteins may regulate virus protein expression in virus-producing cells, which can negatively affect the infectivity of the progeny BmNPV. The roles of the m6A modification in BmNPV-infected cells with respect to RNA splicing and stability remain to be elucidated in additional studies.

Many studies have been carried out to investigate m6A biology in various species. However, for the first time, we profiled the m6A methylome in the silkworm midgut, and compared the distribution characteristics of the m6A modification in mRNAs following BmNPV infection. The distribution of the m6A modification was altered in the midgut transcriptome following BmNPV infection and *in vitro* experiments showed the impact of the expression levels of the m6A machinery on BmNPV infection dynamics. The epitranscriptome data in this work provide an important reference for the study of critical RNA modification in insects and will aid in the understanding of the mechanism and pathogenesis of BmNPV.

## Data Availability Statement

The raw data were submitted to the Sequence Read Archive with accession numbers (SRR10141250, SRR10141249, SRR10141248, and SRR10141247).

## Author Contributions

CG and XH were responsible for conception and design of the study. XZ, YZ, and KD were responsible for data acquisition and analysis and all other authors were responsible for drafting manuscript and figures. RX and GC were responsible for providing general guidance.

## Conflict of Interest

The authors declare that the research was conducted in the absence of any commercial or financial relationships that could be construed as a potential conflict of interest.

## Footnotes

1 ftp://ftp.ncbi.nih.gov/blast/db

2 http://picard.sourceforge.net/

3 http://smart.embl-heidelberg.de/

4 https://web.expasy.org/compute_pi/
