# Supplementary material for: N6-Methyladenosine Level in Silkworm Midgut/Ovary Cell Line Is Associated With Bombyx mori Nucleopolyhedrovirus Infection
Source: Front Microbiol. 2020 Jan 10;10:2988. doi: 10.3389/fmicb.2019.02988 (PMC6965365; doi:10.3389/fmicb.2019.02988)
Supplement: Supplementary file 1 [file Table_1.DOC]

| Gene name | Forward primers (5’-3’) | Reverse primers (5’-3’) |
| --- | --- | --- |
| TIF-4A | GAATGGACCCTGGGACACTT | CTGACTGGGCTTGAGCGATA |
| METTL3 | CCCAATAACCATGAAATAGC | ATGCTGGTGGAACGATTGGT |
| METTL14 | CCCACCAGTAAGGACCAACC | GGTGTTGCAGTTTCCGCAAT |
| YTHDF3 | TCGCCATTACCAAACGTCCA | GTTGTCCAAATGCCGCTGTT |

Supplementary Table 1 The primers were used for real-time PCR assay.
